# Pre-Treatment Assessments to Identify Treatment Components for an Adolescent with Subtype-3 Self-Injurious Behavior

**DOI:** 10.3390/bs15050664

**Published:** 2025-05-13

**Authors:** Kyle W. Dawson, Amanda M. Morris, Tara A. Fahmie, Kortlyn Tawney, Carter Welch

**Affiliations:** 1 Munroe-Meyer Institute, University of Nebraska Medical Center, Omaha, NE 68106, USA; kyle.dawson@unmc.edu (K.W.D.); amanda.morris@unmc.edu (A.M.M.); ktawney@unmc.edu (K.T.); 2 Psychology Department, Midland University, Fremont, NE 68025, USA; carter.welch@midlandu.edu

**Keywords:** automatic reinforcement, competing stimuli, preassessment, self-injury, self-restraint

## Abstract

This case study explores the use of multiple pre-treatment assessments (functional analysis, reinforcer assessment, alternative self-restraint assessment, modified augmented competing stimulus assessment) to inform a comprehensive treatment package for an adolescent male exhibiting Subtype-3 automatically maintained self-injurious behavior (SIB). These assessments guided the development of a treatment package involving continuous access to competing stimuli and alternative self-restraint responses, differential reinforcement of alternative behaviors, and blocking hand-to-head hits across three contexts. The study also details many points of collaboration between the experimenters and the family and other specialists. Results showed decreases in SIB that were accompanied by a reduction in mechanical restraints and access to alternative activities.

## 1. Introduction

Automatically maintained self-injurious behavior (SIB) is a classification of SIB that occurs independent of social contingencies (Vaughan & Michael, 1982). Recent estimates suggest that approximately 31% of all published cases of SIB are maintained by automatic reinforcement (Melanson & Fahmie, 2023). Hagopian et al. (2015) were the first to describe various subtypes of automatically maintained SIB based on patterns of responding that emerged during a pretreatment functional analysis. Three subtypes were distinguished by levels of responding in the test (e.g., no interaction) and control (e.g., play) conditions of a functional analysis, as well as by the presence of self-restraint.

Subtype-1 was marked by high levels of SIB in the test condition (e.g., no-interaction condition) and differentially lower levels of SIB in the control condition, suggesting that reinforcement-based treatments may displace SIB. Subtype-2 was marked by a lack of differentiation between responding in the test and control conditions, suggesting insensitivity to treatments relying on social reinforcers alone. That is, the reinforcement present in the control condition did not seem to compete with the reinforcement produced by the SIB. Finally, Subtype-3 was marked by maintenance of responding in the test condition and the presence of self-restraint. Hagopian et al. hypothesized that, for Subtype-3 SIB, preferred sensory stimulation may be a source of positive reinforcement for SIB, and escape from the aversive stimulation produced by SIB may be a source of negative reinforcement for self-restraint.

Hagopian et al. (2015, 2017) also examined how subtypes of automatically maintained SIB aligned with, and predicted, the efficacy of various treatment components. Taking data from both studies combined, the authors found that 82.6% of cases of Subtype-1 SIB responded well (i.e., had a >80% reduction in SIB) to treatments that incorporated reinforcement only. Moreover, treatment outcomes for individuals displaying Subtype-1 SIB were similar to treatment outcomes for a control group of individuals displaying SIB maintained by social reinforcers (Hagopian et al., 2017). Individuals displaying Subtype-2 SIB showed more treatment resistance compared to the control group, with only 7.1% of cases of Subtype-2 SIB responding well to treatment that incorporated reinforcement only. In other words, Subtype-2 SIB required more intrusive interventions, such as punishment or blocking, to produce positive outcomes. Comparatively little is known about the efficacy of various treatment components in the reduction of Subtype-3 SIB (Hagopian et al., 2015, 2017). For example, in the analysis by Hagopian et al. (2015), treatment outcome data were reported for only 4 of 8 cases of Subtype-3 SIB. Positive treatment outcomes were obtained with the use of restraints or protective equipment, and reinforcement alone was not even attempted in these cases.

Given the lack of information on treatment outcomes for cases of Subtype-3 SIB, it is imperative that researchers continue to evaluate treatment options for individuals who engage in Subtype-3 SIB. Particularly, researchers should evaluate the efficacy of reinforcement-based components to reduce the need for restrictive procedures (e.g., mechanical restraints) in cases of Subtype-3 SIB. Several pre-treatment assessment formats may lend well to this goal. For example, the competing stimulus assessment (CSA; see Haddock & Hagopian, 2020, for a review) involves identifying stimuli that presumably compete with the reinforcer provided by the self-injury. In a CSA, therapists provide free access to various stimuli and collect data on stimulus engagement and SIB to evaluate the effectiveness of the stimuli in decreasing the occurrence of SIB relative to a control condition (i.e., no stimulus). Through the years, experimenters have examined modifications and provided considerations to the CSA to increase the likelihood of identifying high-competition stimuli (Haddock & Hagopian, 2020).

One recommendation in the CSA literature involves using stimuli that “match” the hypothesized sensory reinforcement provided by the behavior (e.g., Clay et al., 2018; Gover et al., 2019; Piazza et al., 1998, 2000). “Matched” stimuli refer to stimuli that produce sensory stimulation matching (or approximating) that produced by automatically maintained SIB. For example, Piazza et al. (2000) found that shampoo on a hard surface was a more efficacious treatment for reducing saliva play compared to access to a puzzle. In a review on enriched environment as a treatment for behavior maintained by automatic reinforcement, Gover et al. (2019) found that environmental enrichment was efficacious for 64.7% of cases when matched stimuli were selected for use in treatment compared to 6.3% efficacy when unmatched stimuli were used. However, it is unlikely that a clinician will know with certainty the type of sensory stimulation produced by SIB. Research on matched stimuli suggests that considering multiple forms of sensory stimulation when selecting stimuli may be valuable in the CSA. For example, a child who engages in head-directed SIB in the form of heading hitting might contact tactile sensation (e.g., pressure or oscillations), visual stimulation (e.g., flashes of light), or auditory input (e.g., sounds from the impact), among others. Therefore, therapists could incorporate stimuli that produce various types of sensory stimulation (e.g., pressure cap or vibrating toys that produce tactile stimulation) to enhance the likelihood of a stimulus effectively competing with SIB.

Researchers recently provided the framework for an augmented competing stimulus assessment (A-CSA) that can be used when the initial CSA does not produce stimuli with high engagement and low SIB (Frank-Crawford et al., 2023; Hagopian et al., 2020). The A-CSA starts with a free access condition to inform baseline levels of competition. Depending on levels of stimulus engagement and occurrences of SIB, modifications to the procedures, such as prompting engagement with the stimulus or blocking occurrences of SIB, can be introduced to increase the likelihood of identifying an effective competing stimulus. The results of Hagopian et al. (2020) and Frank-Crawford et al. (2023) suggested that the A-CSA can be an effective assessment for identifying and establishing additional competing stimuli.

Additional research is also needed on safer alternatives to self-restraint when topographies of self-restraint are not consistently available or are themselves prohibitive (Schmidt et al., 2024). Rooker and Roscoe (2005) sought to better understand the relation between self-restraint and SIB. In a case study, the researchers conducted an initial functional analysis in which restraint materials were not present; their participant engaged in differentially higher levels of SIB in the demand condition. The researchers hypothesized that access to self-restraint may have functioned as reinforcement for SIB during the demand condition (i.e., task prompts competed with self-restraint and escape enabled self-restraint). Preference and competing stimulus assessments were then used to identify preferred and effective “alternative” restraint materials (e.g., neck pillow). Follow-up functional analyses suggested that self-restraint was automatically maintained, SIB was reinforced by access to self-restraint only when alternative restraint materials were unavailable, and continuous access to alternative self-restraint materials reduced SIB. The researchers recommended further study of safe and acceptable forms of self-restraint.

In sum, the previous literature provides several assessments that a clinician can use to help inform a reinforcement-based treatment package for an individual with Subtype-3 SIB; however, no research to date has shown how the combination of pre-treatment assessments can be used to inform a comprehensive treatment package for individuals with Subtype-3 SIB. The purpose of the current case study was to provide a clinical demonstration of the use of multiple pre-treatment assessments to inform such an intervention. These included a functional analysis, a reinforcer assessment, an alternative self-restraint assessment (ASRA), and a modified version of the A-CSA. From the outcomes of these assessments, we evaluated a treatment package consisting of continuous access to a competing stimulus and alternative self-restraint response, differential reinforcement of appropriate behavior, and blocking hand-to-head hits across three distinct daily contexts. This case study shows the benefit of multiple pre-treatment assessments to inform a comprehensive treatment package for the reduction of Subtype-3 SIB.

## 2. Materials and Methods

### 2.1. Participant

Gerard was a 13-year-old male who was referred to a university-based clinic specializing in the assessment and treatment of severe behavior disorders. Gerard’s primary referral concern was SIB in the form of hand-to-head hitting. Prior to his admission, Gerard had received ABA-based services in an early intervention clinic and from a behavioral company providing consultative services by training caregivers and school staff. The caregivers indicated they had seen some improvement in adaptive skills (e.g., functional communication responses [FCRs]), but sustained levels of SIB prompted the caregivers to seek additional services.

Gerard was a warm and affectionate adolescent who enjoyed listening to music, engaging in social interactions, and relaxing in inflatable furniture. He used an eye-tracking speech output device and vocal approximations to communicate his wants and needs. He was also able to answer questions regarding his wants by using eye gaze to indicate preference. Previous providers had taught Gerard to make requests for tangible items and access to the bathroom. Additionally, Gerard had been taught by previous providers to use the vocal approximation or speech output device to request a break as a replacement for what was presumed to be socially mediated SIB. Throughout his admission, we continued to reinforce the break FCR to honor his withdrawal of assent for the treatment and to ensure maintenance of the skill. Gerard’s parents were his primary caregivers and were heavily involved in his therapeutic care. Their input was frequently used to set goals, guide treatment decisions, select session materials, troubleshoot barriers, and evaluate outcomes.

Gerard had received a diagnosis of autism spectrum disorder, attention-deficit hyperactivity disorder (unspecified), a cortical visual impairment (CVI), hypotonia, and a genetic disorder. At the time of admission, Gerard was learning to use a cane for walking and an eye-tracking device for communicating. He required the near-continuous use (including during sleep) of mechanical restraints to keep him safe from SIB.

### 2.2. Setting and Materials

All sessions took place in a university-based clinic specializing in the assessment and treatment of severe behavior disorders. The clinic was equipped with padded session rooms with a one-way mirror for discrete data collection. The clinic also contained a padded restroom. The location of sessions and materials used within each session was dependent on the specific assessment or treatment conditions (see Table 1 for a summary of setting, materials, and personal protective equipment across conditions). The FA, reinforcer assessment, ASRA, A-CSA, and portions of treatment (see below for procedural details) took place in a padded session room equipped with inflatable furniture, a table and chairs, and a stand for the speech generating device. A second padded session room equipped with Gerard’s highest preferred inflatable furniture, a table and chairs for meals, and a stand for the speech generating device was used for breaks. Treatment in the bathroom context took place in a padded bathroom near the session rooms, and transitions took place in hallways leading to various locations (e.g., waiting room, gymnasium, outdoors) throughout the clinic.

Prior to receiving services at our clinic, the caregivers used mechanical restraints to ensure Gerard’s safety, and these restraints were used at different points within his admission at our clinic to promote safety. Specifically, Gerard used a cushioned belt that wrapped around his torso and wrist cuffs that enclosed his wrists and attached to the belt to limit Gerard’s range of motion when engaging in hand-to-head hitting. Thus, wrist cuffs did not fully prevent SIB, but they did significantly decrease its intensity. In conversations with caregivers, decreasing the use of the wrist cuffs was a primary goal in the admission, as the wrist cuffs decreased Gerard’s ability to interact with the environment and limited his ability to catch himself properly if he lost balance while walking. The wrist cuffs were used in parts of the functional analysis (FA), in treatment baseline sessions, and in the bathroom context before they were eventually faded out in all treatment contexts. Upon discharge from the program, Gerard only used the cuffs when caregivers were unavailable to block SIB (e.g., while in a car, when in a different room) and while he was sleeping.

The reinforcer assessment included cards created in consultation with a CVI specialist for target responses and various leisure items as potential reinforcers. In the ASRA, the researchers used different types of clothing, clothing accessories, and sports and fitness materials as mechanisms for alternative self-restraint. Materials in the A-CSA included stimuli that were and were not matched to the potential sensory stimulation provided by SIB. Finally, the treatment package included items from the reinforcer assessment, ASRA, A-CSA, and various stimuli needed for academic instruction and daily living skills. Additional details on these materials are below.

### 2.3. Response Measurement and Definition

The primary dependent variable for all conditions was SIB in the form of hand-to-head hitting. Additional dependent variables used throughout the admission included self-restraint, alternative self-restraint, and stimulus engagement.

Self-injury was defined as forceful contact or attempted contact of Gerard’s hand (open or closed, with or without an object) against his head from a distance of at least 6 inches. Observers scored the frequency of SIB and summarized those data as responses per minute (RPM) by dividing the frequency of SIB by the session duration in minutes. Self-restraint was defined as Gerard placing an uncuffed hand (or hands) underneath his stomach while lying in a prone position on inflatable furniture or sticking his hand underneath of his shirt while wrapping his shirt around his hand. If Gerard was wearing his wrist cuffs and lying on his cuffed hand, observers did not score this response as self-restraint, as Gerard had limited mobility of this arm. The alternative self-restraint response was defined as using one of the stimuli provided in the ASRA, or placing his hands behind his back, to restrict movement and impede SIB. Generally speaking, self-restraint consisted of engaging in a restrictive response that could have its own detrimental effect, whereas alternative self-restraint consisted of engaging in a safer alternative response to restrict SIB. Stimulus engagement was defined as Gerard using his hands to manipulate a stimulus or his body to orient to a visual stimulus that did not require the use of hands. For example, if Gerard was oriented toward the tablet during a video, data collectors scored this behavior as stimulus engagement. Data collectors recorded the duration, in seconds, of self-restraint, alternative self-restraint, and stimulus engagement, and converted the duration of responding to a percentage of session by dividing the response duration by the session duration and multiplying by 100.

### 2.4. Procedures

#### 2.4.1. Safety Protocol

Due to the specific topography of SIB and potential risks associated with the response, we incorporated several components throughout assessments and treatment to mitigate safety risks. First, response blocking was in place during all sessions. The researchers remained within arms-length of Gerard at all times and blocked all attempts to engage in SIB. Additionally, Gerard wore the wrist cuffs at times when the therapist was unavailable to block SIB (e.g., while Gerard was sitting on the toilet).

In addition to the safety modifications described above, we also included procedures to allow for assent-withdrawal from participating in sessions. If Gerard used any form of communication to ask for a break or to return to his highest preferred inflatable furniture, therapists immediately paused the ongoing activity and allowed Gerard to return to the inflatable furniture and engage in self-restraint if desired. The therapists waited until Gerard was engaging with therapists and abstaining from SIB before initiating a transition back to the previous context.

#### 2.4.2. Functional Analysis

Gerard’s clinical team implemented an automatic function screener, similar to that described by Querim et al. (2013), prior to the implementation of a multielement FA. In all test conditions of the FA, unless otherwise noted below, only Gerard’s left hand was placed in his wrist cuffs, as this was reported to be his preferred method of relaxing on the inflatable furniture. Gerard’s right hand was not placed in the wrist cuffs, which still allowed for the opportunity to engage in, and therefore evaluate, SIB.

In the minimal-interaction test condition^1^, therapists were present in close proximity to Gerard but did not interact with Gerard except to block SIB as described above. Gerard did not have access to any items besides the inflatable furniture, which he laid on in a prone position throughout this and all phases of the FA. The attention condition was identical to the minimal interaction condition except that the therapists provided 30 s access to attention in the form of physical attention (e.g., tickles, back scratches) and high-energy vocal interactions that were reportedly preferred (e.g., therapist dancing while interacting with Gerard) contingent on each instance of SIB. In the tangible condition, therapists provided presession access to music via a tablet and access to fidget items. Once Gerard was engaged with the tangible items, the therapist removed the items and did not interact with Gerard. Contingent on each occurrence of SIB, the therapists provided access to the tangible items for 30 s. In the play condition, therapists provided continuous attention and free access to music and fidget items. Self-injury did not produce any differential consequences. In the escape condition, therapists used a three-step guided prompting strategy to assist Gerard in completing gross motor tasks, which required him to momentarily disengage in self-restraint. Contingent on each occurrence of SIB, the therapists informed Gerard that he could have a break and provided a 30 s break from instructions. Due to high rates of SIB in the escape test condition, the research team implemented a self-restraint interruption condition to evaluate whether responding in the escape condition was maintained by escape from instructions or access to self-restraint. In the first self-restraint interruption session and subsequent play session, both of Gerard’s hands were removed from the wrist cuffs. Due to high rates of SIB in these sessions, therapists placed his left hand back in the safety cuff in subsequent sessions. At the beginning of self-restraint interruption sessions, the therapists removed Gerard’s hand (or hands) from his self-restraint, placed his hand(s) on the inflatable furniture, blocked access to attempts to reinitiate self-restraint, and provided no other instructions. Contingent on SIB, the therapist stopped blocking self-restraint and provided free access to self-restraint for 30 s. In this way, the self-restraint interruption condition was similar to the escape condition in that Gerard’s hands were removed from self-restraint and self-restraint was available during reinforcement. The key difference between the two conditions was the presence and absence of instructions. Therefore, escape from instructions was not a putative reinforcer for SIB in the self-restraint interruption condition.

#### 2.4.3. Reinforcer Assessment

A reinforcer assessment was conducted to identify the reinforcing efficacy of various stimuli when presented contingent on a simple response. Initially, all reinforcer assessment conditions took place with Gerard seated on a bench at a table. In the FR1 eye gaze condition, therapists provided 30 s of access to the selected reinforcer for the occurrence of a target response (i.e., looking at a black card with a red circle) informed by the CVI specialist. This target response was selected due to caregiver interest in strengthening skills needed to use Gerard’s eye tracking device for communication. Following 30 s access, the therapists removed the item and waited for Gerard to engage in another response. Therapists continued to reinforce all target responses on a fixed-ratio 1 (FR1) schedule of reinforcement for the duration of the 5 min session. In the control condition, therapists provided Gerard with continuous access to all the reinforcers used in the test conditions. Opportunities to engage in the target response were available, but occurrences of the target response did not produce differential consequences.

After initially evaluating the effectiveness of the different stimuli, the authors selected the top two tangible reinforcers (i.e., music and beads) and the top attention type (i.e., ear squeezes) to use while simultaneously evaluating their effectiveness when combined with a preferred seating arrangement (i.e., inflatable chair). The purpose of this condition was to evaluate the reinforcing efficacy of the items on increasing alternative behavior while using the inflatable furniture as a potential competing item. Test and control conditions were otherwise identical to those used in the FR1 eye gaze condition. This evaluation was conducted following the ASRA (see below).

Finally, a second response that was not in Gerard’s repertoire was selected to evaluate the efficacy of the reinforcers in teaching a new response—a card touch. This target response was selected by the clinical team as a common target of skill acquisition programs. Therapists used a progressive delay to a physical guidance prompt to teach Gerard to engage in the target response. The therapists gave the initial instruction (e.g., “Touch the card”) and then immediately provided physical guidance. After two consecutive sessions with SIB below the mean rate of responding in the escape condition of the FA, the therapists increased the prompt delay. Therapists provided 30 s of access to the pre-programmed reinforcer contingent on both prompted and independent card touches. In the control condition, the therapists provided continuous access to the reinforcers used in the test condition and provided the instruction, “Touch the card” every 30 s to match the density of instructions used in the test condition. Card touches in the control condition produced no differential consequences.

#### 2.4.4. Alternative Self-Restraint Assessment (ASRA)

The research team conducted an ASRA to identify alternative forms of self-restraint that Gerard could engage in instead of engaging in the more limiting form of self-restraint of lying in a prone position on his hands. In the ASRA, therapists provided Gerard with access to different items that he could use to engage in alternative forms of self-restraint, and the items were quasi-randomly rotated such that all items were presented once before any items were repeated.

Prior to the onset of each session, therapists conducted five forced-exposure trials by physically guiding Gerard to engage in alternative self-restraint. After the final forced-exposure trial, the therapists ensured Gerard was engaging in the alternative self-restraint response and then started the session. During the sessions, therapists did not prompt Gerard to engage in alternative self-restraint and did not provide consequences (e.g., attention, tangible items), except for blocking SIB, for the alternative self-restraint response. Sessions were initially 2 min in duration. After effective alternative self-restraint responses were identified, session duration was increased to 5 min to evaluate the efficacy of the responses in longer intervals.

A condition consisting of continuous restraint in the wrist cuffs served as a control. In the control condition, Gerard was placed in the wrist cuffs, and therapists remained close to Gerard to block any attempted SIB. Because the wrist cuffs were considered the most effective way to maintain safety (while still allowing attempts and lower-intensity SIB to occur), we used this as a control condition so that we could evaluate if any alternative self-restraint responses were as effective as the wrist cuffs in maintaining Gerard’s safety. We did not include a no-restraint control condition (a more standard control) due to safety risks inherent in doing so with the high levels of SIB exhibited by Gerard.

#### 2.4.5. Modified Augment Competing Stimulus Assessment (A-CSA)

A modified A-CSA was used to identify items that could compete with SIB. We note that our version of the A-CSA was modified from the procedures described by Frank-Crawford et al. (2023) and Hagopian et al. (2020) in that response disruption (i.e., blocking SIB) was present from the onset of the A-CSA to mitigate the risk of injury produced by SIB. Although not a condition described in previous studies, we used the response disruption condition as the first condition instead of the free access condition (which does not involve response disruption) used previously. In addition, we did not repeat a response disruption condition in the same manner that researchers have previously repeated the free access condition. Therefore, our modified CSA consisted of two conditions: (a) response disruption and (b) response promotion and disruption.

Prior to the assessment, the therapist demonstrated how to use each item by physically guiding him to engage with the item for approximately 10 s. Gerard was seated in an inflatable chair without the use of the wrist cuffs, but Gerard had free access to his alternative self-restraint responses (hand warmer, hands behind back) identified in the ASRA during the entirety of the session.

In both conditions, the therapist provided access to each item sequentially and recorded data on the frequency of SIB and duration of item interaction. If Gerard dropped an item during the session, the therapist waited 5 s and then returned the item following the first instance of him dropping the item. If Gerard dropped the item a second time, the therapist did not replace the item unless Gerard engaged in some type of indicating response towards the item (this did not happen during the evaluation) or met the criteria for prompted engagement (see below for details). All sessions of the A-CSA were 5 min.

In the response disruption condition, the therapist blocked all attempts of SIB to ensure safety and to reduce access to potential sensory reinforcers. The therapists did not prompt Gerard to interact with the item nor provide any attention following blocked attempts. In the response promotion and disruption condition, the therapists continued to block all occurrences of SIB while also redirecting Gerard to engage with the item. If 10 s elapsed without item engagement, the therapist would prompt Gerard to interact with the item by saying “Gerard, you can play with the [toy] like this!” and physically guiding Gerard to play with the item for at least 5 s. Additionally, if Gerard attempted to engage in SIB, the therapist would block the attempt and then redirect Gerard’s hands to the item and prompt him to engage with the item.

#### 2.4.6. Comprehensive Treatment Package Evaluation

Following the pre-treatment assessments, therapists designed a reinforcement-based treatment package to reduce the frequency of SIB using the results of the reinforcer assessment, ASRA, and A-CSA. A multiple baseline across contexts design (with embedded reversal design in one context) was used to evaluate the effectiveness of the treatment package when Gerard was expected to complete or tolerate tasks.

The tasks in each context were context-specific. In the chair context, tasks were informed by caregiver endorsement, and initially involved tolerance of daily living tasks (e.g., wiping his face). After sustained success in daily living tasks, caregivers endorsed attending to academic tasks, such as digital books provided and created by caregivers and previous providers, as appropriate instructions in the chair context. In the bathroom context, Gerard was expected to tolerate and participate in tasks related to a toileting routine (e.g., sit on the toilet, wash hands). In the transition context, tasks included walking to locations and touching visual stimuli endorsed by caregivers and the CVI specialist that were associated with locations. Aside from context-specific instructions, the procedures were identical across the different contexts. The only exception was the continued use of the wrist cuffs while Gerard sat on the toilet in the bathroom context only.

In the baseline for all contexts, Gerard’s hands were removed from the wrist cuffs, and therapists provided a variety of context-relevant instructions (e.g., turning on water in the bathroom context, gross motor instructions in the chair context) continuously throughout the session, unless Gerard engaged in an FCR for a break. Therapists used a graduated guidance prompting procedure to prompt Gerard to complete instructions and blocked all attempted occurrences of SIB. Therapists did not provide a break or access to preferred stimuli contingent on SIB, but rather blocked any instances and continued with instructions. Again, therapists reinforced all FCRs for a break or to return to his preferred inflatable furniture. Therapists provided verbal attention during breaks (e.g., praise for sitting), but physical attention and preferred items were not presented during breaks. Additionally, if Gerard initiated the next step of the routine before 60 s elapsed, therapists allowed him to continue with steps but did not provide instructions until 60 s elapsed.

The treatment package consisted of differential reinforcement of appropriate behavior (i.e., compliance or tolerance of tasks; DRA), continuous access to competing stimuli, continuous access to alternative self-restraint, and response blocking and redirection to alternative self-restraint contingent on attempted SIB. We included a vibrating toothbrush and swim cap as competing stimuli, access to beads and preferred music/tablet as reinforcers for compliance with tasks, and the hand warmer and hands behind the back as alternative self-restraint options.

Prior to issuing instructions, the therapists provided Gerard access to at least one competing stimulus and the hand warmer. Contingent on compliance with or tolerance of tasks in the bathroom context and chair context, therapists provided 60 s access to the reinforcers on a FR1 schedule of reinforcement. Due to Gerard’s visual impairment and hypotonia, we determined that pausing transitions for reinforcement could be unsafe in the transition context; therefore, we provided noncontingent access to attention during the transition and contingent access to his reinforcers immediately following the transition.

In the bathroom and chair context, the schedule of reinforcement was thinned in sessions 19 and 161, respectively. In the bathroom context, the schedule was thinned from an FR1 schedule (i.e., reinforcer delivered following completion of each instruction) to a schedule in which reinforcement was provided following the completion of the entire bathroom routine (approximately a variable-ratio five [VR5] schedule of reinforcement). In the chair context, the schedule of reinforcement was thinned to a variable-interval 2 min (VI2) and coincided with a change in tasks. Specifically, to better equip Gerard for a transition back to school, we modified the tasks presented in the chair context to be academic based. Additionally, we evaluated the feasibility of fading the proximity of the second (blocking) therapist in the chair context. Prior to conducting the academic instruction baseline, the second therapist was placed at their original proximity per caregiver request. Finally, the use of the swim cap as a competing stimulus was discontinued at session 174 due to Gerard frequently removing the swim cap by rubbing his head on his shoulder or wall behind the toilet.

## 3. Results

### 3.1. Functional Analysis

**Figure 1:**
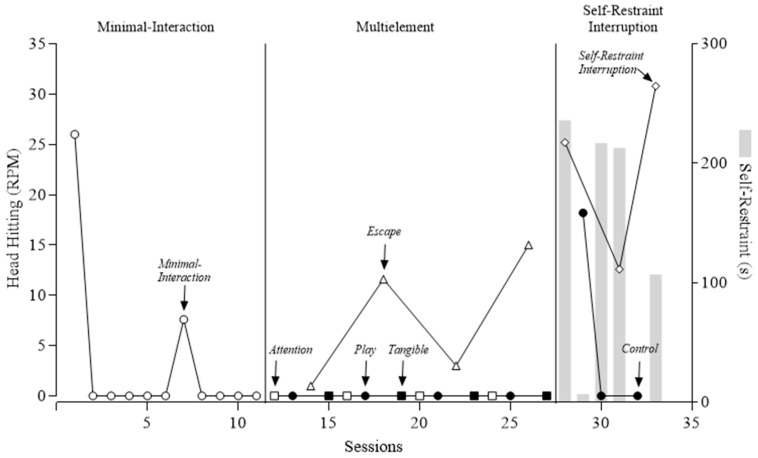
Functional analysis of self-injurious behavior. RPM = responses per minute. Gray bar indicates duration of self-restraint (in seconds).

Figure 1 shows the outcomes of Gerard’s FA. In the screener for automatic reinforcement (i.e., minimal-interaction test condition), SIB occurred in only two sessions. As a reminder, Gerard wore one wrist cuff and was lying in a prone position on his hand or hands (i.e., self-restraint) during this phase. Although we did not initially collect data on self-restraint, it can be anecdotally reported that the variability in this phase was primarily due to the presence and absence of self-restraint with his uncuffed arm. Specifically, Gerard was consistently lying on the cuffed arm but would also engage in self-restraint with the uncuffed arm by placing that arm underneath his body in the prone position. He did not engage in SIB when engaging in self-restraint with his uncuffed arm, but elevated levels were observed when Gerard was not engaging in self-restraint. As a result, we made a preliminary hypothesis that the function of SIB was best classified as Subtype-3 automatic reinforcement.

We next conducted a test of attention, tangible, and escape conditions to determine whether any social contingencies were likely to exacerbate SIB when self-restraint was available. In this multielement series, Gerard engaged in higher levels of SIB during the escape condition compared to all other test and control conditions. This suggested a secondary escape function. However, we were unable to determine if the increase in SIB was evoked by the instructions or by self-restraint being interrupted to prompt a gross motor response.

Thus, to confirm a Subtype 3 classification, we conducted the self-restraint interruption condition, during which we collected data on self-restraint while no demands were presented and Gerard’s arm was removed from underneath him for brief periods of time. We observed elevated rates of SIB at or above those shown in the previous escape condition. Additionally, Gerard engaged in high and variable durations of self-restraint (mean of 43% of session time across both self-restraint interruption and control sessions, as indicated by the gray bars in Figure 1). Given the presence of self-restraint and high levels of SIB when self-restraint was blocked, we gained more confidence that SIB was maintained by automatic reinforcement (i.e., we ruled out social reinforcement) and that access to self-restraint decreased its occurrence (i.e., Subtype-3 SIB).

### 3.2. Reinforcer Assessment

**Figure 2:**
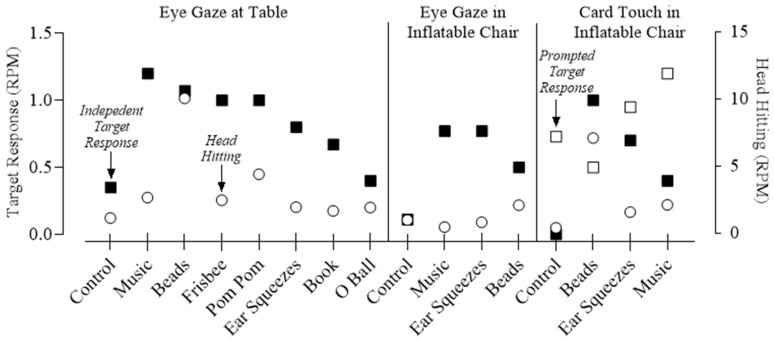
Outcomes of the reinforcer assessment. RPM = responses per minute.

Figure 2 shows the outcomes of our reinforcer assessment, collapsed across 67 sessions total (27 sessions in phase 1; 26 sessions in phase 2; 14 sessions in phase 3; session-by-session data available upon request). When all putative reinforcers and attention were provided noncontingently in the control condition, independent target responding (eye gazing or card touching) occurred at rates of 0.35, 0.11, and 0.00 RPM across respective phases. Self-injury remained relatively low during the control condition, with a rate of 1.15, 1.0, and 0.42 RPM across respective phases. In phase 1, putative reinforcers were provided contingent on eye gazing while Gerard was seated at a table. Target responding occurred at a higher rate than in the control condition (0.35 RPM) when music (1.20 RPM), beads (1.07 RPM), frisbee (1.0 RPM), pom pom (1.0 RPM), ear squeezes (0.8 RPM), book (0.67 RPM), and O-ball (0.40 RPM) were delivered contingent on eye gazing. However, rates of SIB were also higher with all items (range = 1.67–10.06 RPM). In phase 2, putative reinforcers were provided contingent on eye gazing while Gerard was seated in an inflatable chair. The change in seating was an attempt to reduce SIB during the assessment. We included only music, beads, and ear squeezes in this phase; these items were selected based on their strength as reinforcers or their ease of implementation (see extended rationale below). During phase 2, target responding occurred at a higher rate than in the control condition (0.11 RPM) for all three items: music (0.77 RPM), ear squeezes (0.77 RPM), and beads (0.5 RPM). In addition, SIB remained relatively low across the phase (range = 0.47–2.1 RPM). Finally, we increased the effort involved in the target response by prompting a card touch in phase 3. Again, we tested only the music, ear squeezes, and beads in this phase, and they again produced independent target responding at higher levels than the control (control = 0.0 RPM, beads = 1.0 RPM, iPad = 0.4 RPM, ear squeezes = 0.7 RPM). In addition, prompted responses occurred at a higher rate when music and ear squeezes were delivered. However, SIB occurred at a higher rate (range = 1.58–7.11 RPM) during the test conditions of this phase compared to the control (0.42 RPM).

Based on these aggregated data, along with caregiver consultation, music, ear squeezes, and beads were used as reinforcers. These three items were selected over a few items with higher target rates (e.g., frisbee, pom pom) in the first phase of our assessment due to the practicality of their delivery and endorsement by caregivers. Gerard could passively engage in ear squeezes and music without any motor requirements. Given that Gerard had physical limitations, this passive consumption of reinforcers was endorsed as a priority by caregivers. Although the beads did require motor skills (e.g., grabbing, holding), Gerard’s caregivers endorsed this selection due to Gerard’s reported preference for manipulating beads and mastery of the motor skill involved in tangling his hands in the beads. By contrast, the frisbee and pom poms were more challenging for Gerard to keep in his hands without dropping. Finally, there was no decreasing trend in target responding across sessions of phase 1 when music, beads, and ear squeezes were delivered, whereas there were decreasing trends with some of the other items (frisbee, book, o-ball; data available by request).

### 3.3. ASRA

**Figure 3:**
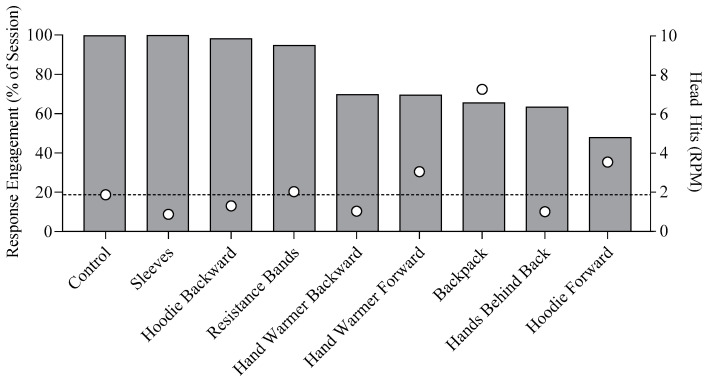
ASRA outcomes. RPM = responses per minute. The dashed line indicates the level of SIB in the control condition.

Figure 3 shows the outcomes of our ASRA, collapsed across sessions (10 sessions of control, 3 sessions of sleeves, 7 sessions of hoodie backward, 15 sessions of resistance bands, 9 sessions of hand warmer backward, 11 sessions of hand warmer forward, 10 sessions of backpack, 3 sessions of hands behind back, and 11 sessions of hoodie forward; session-by-session data available upon request). When the wrist cuffs were used (control), SIB occurred at a rate of 1.87 RPM, and engagement was 99.8%^2^. Other items assessed included the compression sleeves, hoodie backward, resistance bands, hand warmer backward, hand warmer forward, backpack, hands behind the back, and hoodie forward, which produced engagement at 100%, 98.3%, 94.9%, 69.8%, 69.7%, 65.7%, 63.4%, and 48.2%, respectively. SIB occurred at a rate of 1.87 RPM in the control condition, where wrist cuffs drastically inhibited the force of any successful head hit. Several alternative self-restraint items produced SIB rates close to or lower than the rates produced by the cuffs, including sleeves (0.87 RPM), hoodie backward (1.3 RPM), resistance bands (2.03 RPM), hand warmer backward (1.03 RPM), and hands behind back (1.0 RPM). The hand warmer forward was associated with slightly elevated rates of SIB (3.05 RPM), and the backpack was associated with the highest rates of SIB (7.27 RPM). Compared to a condition in which no self-restraint or control items were available (i.e., the control condition of the A-CSA and the self-restraint interruption condition of the FA; see above), however, SIB was reduced across all items included in this assessment.

As with other assessments, our selection of items to include in the treatment comparison was guided both by data from the assessment and by caregiver input. In this case, caregivers considered the accessibility and comfort of the alternative self-restraint response, along with its perceived likelihood of stigmatization. For accessibility purposes, the caregivers preferred the “hands behind back” response, consisting of teaching Gerard to place his arms behind his back while seated. This response did not require any additional equipment, which could become lost or damaged. This also best mimicked Gerard’s preferred self-restraint response (lying in the prone position with hands under the stomach) without prohibiting Gerard’s participation in a variety of activities, such as educational sessions, while seated.

However, the caregivers also wanted an alternative self-restraint response that would be accessible while standing upright and walking. They indicated a preference for the hand warmer placed on his body in the forward position for a few reasons. First, it was less likely to be stigmatizing, as it blended in with his clothes and did not appear overly restrictive when in use. It also allowed Gerard to move his hands in and out at his will. Unlike the compression sleeves, hoodie backward, and resistance bands, Gerard had the motor skills necessary to engage and disengage with the hand warmer, although modifications (i.e., widening the opening to the hand warmer, a modification made at session 44 of the reinforcer assessment) were required to improve his ability to independently engage with the hand warmer. Other items required the assistance of a therapist to initiate self-restraint and could not be disengaged at will (as evidenced by high engagement data). Thus, although engagement was relatively higher with some items, those items were not selected because Gerard was not as independent with their engagement.

### 3.4. Modified A-CSA

**Figure 4:**
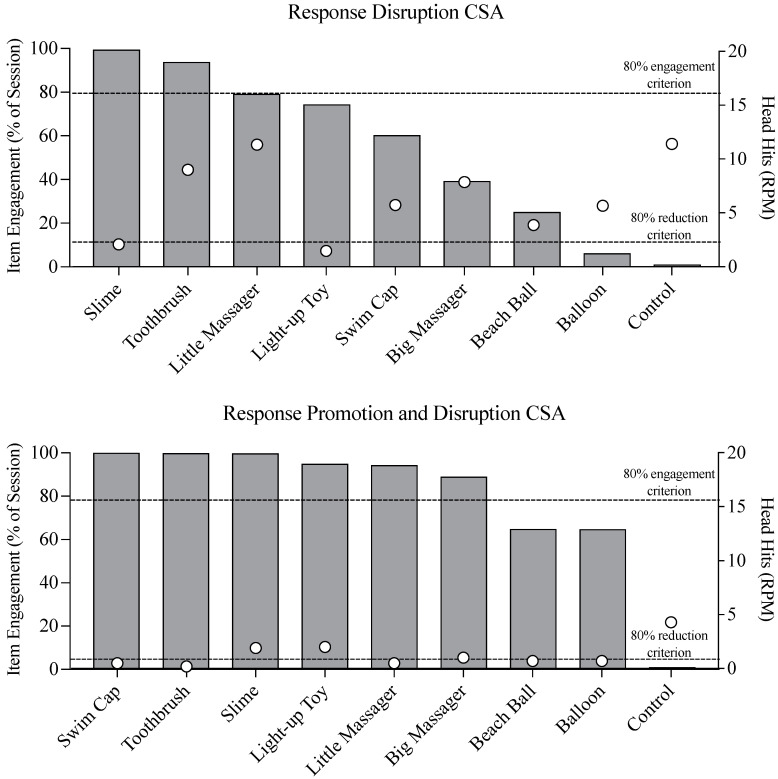
Response disruption CSA (top panel) and response promotion and disruption CSA (bottom panel). CSA = competing stimulus assessment, RPM = responses per minute.

Figure 4 summarizes the outcomes of Gerard’s A-CSA. Data on the top panel of this figure are an average of three sessions of free access to putative competing stimuli with response disruption and are organized by percent item engagement. Gerard engaged with items to varying degrees, with slime at the high end (99.5% average engagement) and balloon at the low end (6.2% average engagement). Higher levels of item engagement were not always associated with lower levels of SIB, but each item was associated with lower levels of SIB compared to the control trial (i.e., no item). However, only one item (light-up toy) was associated with a greater than 80% reduction in SIB below control levels, as depicted by the bottom dashed line on Figure 4. Therefore, we added prompting to the A-CSA and reevaluated the extent to which item engagement and SIB covaried.

The bottom panel of Figure 4 displays data on item engagement and SIB when prompting (i.e., response promotion) was added to the A-CSA. Under these conditions, item engagement remained at near ceiling levels for 6 of 8 putative competing stimuli, and SIB remained below the 80% reduction line for three of those items (swim cap, electric toothbrush, little massager). In consultation with Gerard’s family on the practicality of extended access to these three items, we selected the swim cap and toothbrush for our treatment package. The toothbrush was selected because it had a vibrating feature, similar to the little and big massagers, but could more easily be accessed by Gerard at will by placing it on a lanyard around his neck, which the team did after the A-CSA. Additionally, holding the toothbrush to access the sensory stimulation also served as an incompatible response when he was pressing the toothbrush against his torso, which was a common method he used to interact with the stimulus. The swim cap was selected because it could be worn unobtrusively throughout the session.

### 3.5. Comprehensive Treatment Package Evaluation

**Figure 5:**
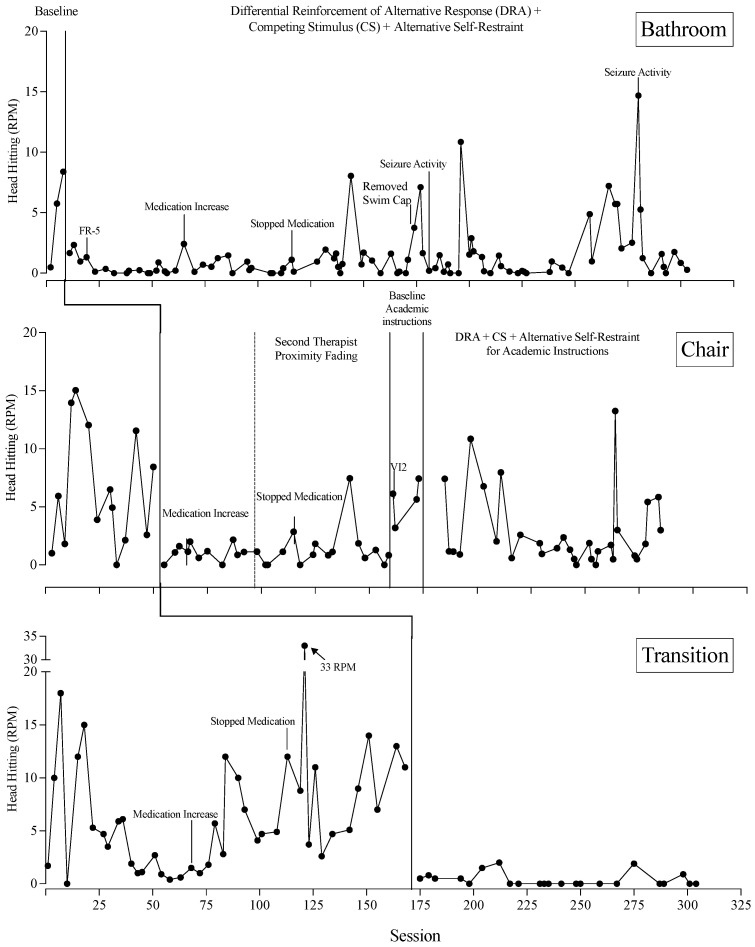
Treatment evaluation across three contexts. CS = competing stimuli, DRA = differential reinforcement of alternative behavior, FR = fixed ratio, VI = variable interval.

Figure 5 depicts the outcomes of Gerard’s treatment program evaluation. Treatment implementation was staggered across three baselines: (a) bathroom context, where SIB occurred at a mean rate of 4.88 RPM in baseline; (b) chair context, where SIB occurred at a mean rate of 6.42 RPM in baseline; and (c) transitions context, where SIB occurred at a mean rate of 6.70 RPM in baseline. The addition of the treatment package resulted in an overall reduction in SIB by 71.4% in the bathroom context (*M =* 1.40 RPM), 63.6% in the chair context (*M =* 2.34 RPM), and 94.6% in the transition context (*M =* 0.37 RPM).

Specific to the bathroom context, SIB steadily increased during baseline and immediately reduced to low and steady rates upon the introduction of the treatment package. Medication was increased and discontinued at two points during treatment, but this did not have a noticeable effect on SIB in the bathroom context. At session 171, the swim cap was removed as a competing stimulus. This change was made in response to Gerard’s repeated attempts to remove it. Although this seemed to have a negative effect on behavior during the 2–3 sessions that followed the change, SIB quickly reduced back to low levels thereafter. Seizure activity occurred at sessions 181 and 280 and was accompanied by elevations in SIB, but the patterns of responding were not consistent across the two documented seizures; thus, it is difficult to confirm whether SIB rates were temporarily influenced by the seizures. The average rate of prompting the alternative self-restraint response in this context was 0.13 per min.

Specific to the chair context, SIB occurred at variable but elevated rates during the baseline phase. An immediate reduction in SIB occurred upon the introduction of the treatment package. These reductions were mostly maintained even when the second therapist faded their proximity to Gerard. However, rates were relatively elevated when a new task (academic instructions) was introduced under baseline conditions. The treatment package was next implemented with academic instructions at the chair, and despite the fact that an overall reduction occurred relative to the baseline condition, there was considerable variability in this final phase. The average rate of prompting the alternative self-restraint response in this context was 0.67 per min.

Specific to the transition context, SIB occurred at variable rates during the baseline phase, ending on a slightly increasing trend before the treatment package was introduced. During baseline, an increase in medication on session 68 was perhaps responsible for an increasing trend in SIB, but this effect was not reversed when medication was discontinued on Session 113. An immediate and sustained reduction in SIB occurred following the introduction of the treatment package. No prompts to engage in the alternative self-restraint response occurred in this context.

## 4. Discussion

SIB that co-occurs with self-restraint (Subtype-3 classification) has proven particularly challenging to treat in the limited research that exists (Hagopian et al., 2015). That is, reinforcement alone has not been documented to result in clinically significant (80% or greater) reductions in this subtype of SIB, and blocking, punishment, and restraints are more often attempted with this subtype than others (Hagopian et al., 2015, 2017). Unfortunately, treatment packages (including both reinforcement and more intrusive procedures) are rare and not well documented for this subtype, leaving clinicians with minimal guidance and researchers with extra motivation to explore innovative approaches. The current study was one such evaluation, in which a novel packaged intervention was shown to reduce the occurrence of Subtype-3 SIB in an adolescent male across three contexts in the clinical setting.

The current study extended existing research on Subtype-3 SIB in several important ways. Most notably, we evaluated a unique treatment package informed by several experimental assessments, including an FA, a reinforcer assessment, an ASRA, and an A-CSA. We identified treatment components that would compete with both the putative appetitive and aversive properties of SIB that are generally hypothesized to contribute to the development of Subtype-3 SIB (Hagopian et al., 2015). The result was a treatment package that not only reduced automatically maintained SIB, but also successfully reduced the use of restrictive mechanical restraints (wrist cuffs) and a prohibitive form of self-restraint (lying on hands in a prone position). Although formal data on the latter were not collected, procedural features of our treatment package (i.e., removing the wrist cuffs, sitting in a chair) eliminated Gerard’s opportunity for mechanical restraint and prohibitive self-restraint.

Although our design did not permit us to distinguish the relative contribution of each treatment component in our package, it is important to consider the behavioral mechanisms potentially underlying them. We hypothesize that noncontingent access to competing stimuli, as identified in our A-CSA, served to either replace sources of automatic positive reinforcement derived from SIB (e.g., head pressure from swim cap replacing head pressure from hitting) or compete with those same sources (e.g., rocking in the inflatable chair competing with stimulation to the head). Additionally, engagement with some of the competing stimuli (e.g., pressing vibrating toothbrush against the torso with hand otherwise used for SIB) may have inadvertently reinforced incompatible behavior through a process of either positive reinforcement (e.g., vibration), negative reinforcement (e.g., avoidance of pain produced by SIB), or both. Specific forms of competing item engagement were not scored in the current case study, and this presents an avenue for future research. That is, it may be helpful to identify topographies of competing stimulus engagement during the A-CSA to better ascertain the likely mechanisms responsible for automatically maintained SIB reduction.

Our A-CSA results suggested that prompting was a critical component in identifying a wide array of potential competing stimuli. Prompting increased engagement to 80% or greater with four of the seven stimuli that had engagement below 80% in our unprompted condition. The prompted condition was also associated with substantially lower rates of SIB for all tested stimuli. One limitation of our analysis is that we did not return to an unprompted condition following our prompted condition; thus, it is unknown whether prompting, per se, or time in the assessment resulted in these changes to engagement and SIB. In addition, our protocol deviated from that described by Hagopian et al. (2020) in a few ways. First, we did not conduct a free access condition without also including aspects of response “disruption” (i.e., blocking SIB). Second, we did not repeat our initial condition to identify whether continued prompting was necessary to sustain high levels of engagement with the stimuli. The decision to conduct the A-CSA using only response disruption and response promotion and disruption conditions was based on our safety protocol (which included blocking when possible) and our desire to expedite the assessment. However, we recognize the benefits of conducting the full A-CSA as described by Hagopian et al. (2020) and recommend adherence to that protocol when possible.

The differential reinforcement contingency included in our treatment package may have strengthened task-related behavior that was only partially compatible with SIB, thereby further reducing attempts to engage in SIB. Deriving stimuli from a reinforcer assessment, as opposed to a preference assessment, provided the clinical team with information on the strength of each stimulus to support more complex multi-response topographies (e.g., adaptive living skills) in a format that was accessible given the participant’s visual impairment. That is, potentially reinforcing stimuli were tested one at a time using a card that was modified based on CVI guidelines; unlike conventional preference assessment formats (e.g., multiple-stimulus-without-replacement format), our reinforcer assessment allowed the participant to access stimuli without visually scanning an array and distinguishing among various stimuli. Outcomes of our reinforcer assessment provided evidence that an eye gaze and a card touch could be strengthened using both social (ear squeezes) and tangible (iPad and beads) reinforcers. We presumed these same stimuli served as reinforcers during the intervention as well, supporting our participant’s behavioral acquisition goals across three contexts. However, we did not conduct a reversal in which reinforcing stimuli were removed, so the function of these stimuli on Gerard’s behavior remains speculative.

Finally, alternative forms of self-restraint (hands behind back, hands in hand-warmer) were prompted during the treatment evaluation in an effort to replace the more restrictive form (lying on hands in prone position) exhibited upon admission to the clinic. Self-restraint is not well understood but is presumably negatively reinforced by avoiding SIB. For Gerard, the alternative forms of self-restraint may have served this function while also being more accessible and efficient than lying in a prone position on his hands. That is, our self-restraint assessment identified topographies that were available when standing, walking, or sitting, and also could be used while engaging in other tasks (e.g., placing one hand in a hand warmer while using the other hand to pick up items). By comparison, lying in the prone position was prohibitive to Gerard’s engagement with tasks that required an upright position (e.g., feeding) or mobility (e.g., walking to the bathroom). Throughout our study, the treatment was applied to various seating arrangements (e.g., a chair, a bench, an inflatable chair, a swing) and transitions to many locations of the clinic (e.g., transitions to the lobby, outdoors, gym, etc.). The variety of positions and locations accommodating our treatment provides evidence of the improved flexibility of the alternative self-restraint response compared to the initial self-restraint response. Although data on the participant’s use of the alternative self-restraint response suggests that it occurred consistently in the absence of consequences, assuming it was a reinforcer is premature based on our prompting criteria. That is, we physically guided the alternative self-restraint response during treatment when SIB occurred. We attempted to encourage independent use of one alternative self-restraint response by decreasing the effort involved in placing hands in the hand warmer, but it is unclear what effect this had on unprompted use of this response, given the limitations of our data collection system.

This study also provides an example of the benefit of collaboration with various stakeholders. Many treatment decisions were informed by *both* the data obtained *and* contributions from the participant’s caregivers and other professionals (e.g., CVI specialist, physical therapist). In some cases, stakeholder input changed a decision that would have been made with data alone. For example, a vibrating toothbrush was selected for use as a competing stimulus due to an accommodation (it could be worn around the neck) recommended for visual impairment, even though it evoked slightly higher levels of SIB than some other items. In addition, the hand warmer was selected by the caregiver as a self-restraint response instead of the sleeves or backward hoodie because the hand warmer looked like a typical clothing item (i.e., less potential for stigmatization) and required relatively fewer fine motor skills to use independently (i.e., more feasible). Generally, all stimuli selected for use during treatment predicted performance better than the control condition (no treatment); however, it remains unknown whether selection of stimuli that performed better during the assessments would have resulted in more rapid or consistent treatment effects across contexts. Nevertheless, we felt it important to plan treatment components that would be both efficacious and socially valid to produce long-lasting and generalizable changes. Although formal data on the social validity of the procedures and generality were not collected following discharge, the team did continue with regular follow-up appointments with the family. During these appointments, the caregivers continued to implement the procedures and were taught how to conduct various assessments (i.e., CSA, ASRA) on an ongoing basis, ensuring the treatment package could be continually adapted as Gerard’s interests evolved with his growth and development.

Given the promising outcomes of this case study, we encourage replication of research aligning preassessments with treatment packages for reducing Subtype-3 SIB. Although our single case study provided preliminary support for the approaches contained herein, we caution that its generality was not established. Thus, we encourage a systematic evaluation of our procedures across consecutively enrolled patients referred for automatically maintained SIB (see Hagopian, 2020, for further discussion). In addition, we encourage the use of standardized protocols, such as the A-CSA (e.g., Hagopian et al., 2020), when possible, as well as a priori criteria (including both data-based and caregiver-informed components) for selection of items to include as reinforcers, competing stimuli, and competing responses in a treatment package. That is, we continue to support involvement of various stakeholders (caregiver, patient, professionals from other disciplines) when possible; however, we recommend using a more formal system (e.g., questionnaire) for collecting their perspectives to enable a clearer summary of factors influencing clinical decision making. To establish the maintenance of the treatment protocol, we encourage data collection across a longer period of time. We also recommend that future researchers collect duration data on self-restraint (assessment and treatment phases) and prompted/unprompted alternative self-restraint responses (treatment phase only) to allow for closer inspection of trends over time and aid in conceptualizations of self-restraint as a potential negative reinforcer. Finally, we recommend including measures of both self-restraint and adaptive behavior in all trials of the ASRA to provide further support for the superiority of the alternative self-restraint response compared to self-restraint.

## Figures and Tables

**Table 1:** Summary of assessment features across conditions of the current analysis.

Assessment	Condition	Setting	Materials	PPE
Functional Analysis	Minimal-interaction, Attention, Play, Escape, and Self Restraint Interruption	Padded session room with client on inflatable furniture with one staff sitting next to him	Inflatable furniture, tablet, fidget, magazine, laptop computer	Wrist cuffs (left hand), blocking pads
Reinforcer Assessment	Fixed Ratio (FR) 1 Eye Gaze and Control	Padded session room with client on bench and one staff on either side of him. Later moved to client sitting in inflatable chair with two staff on either side of him	Laptop computer, bench, eye gaze device, pom pom, beads, O ball, frisbee, book, iPad, inflatable chair	Blocking pads
ASRA	Noncontingent Access to Alternative Self-Restraint	Padded session room with client on bench and one staff on either side of him	Bench, hoodie, wrist weights, resistance bands, backpack, eye gaze device, inflatable furniture, laptop computer	Wrist cuffs (both hands; control sessions only), blocking pads
A-CSA	Response Disruption, Response Promotion and Disruption	Padded session room with client sitting on an inflatable furniture and one staff on either side of him	Laptop computer, pom poms, beads, light-up wand, O balls, inflatable chair	Blocking pads
Treatment	Bathroom Context	Session started in a padded session room. Participant then transitioned to a padded bathroom with one therapist sitting in front of the client	Laptop computer, spare clothes, handwarmer, competing items (e.g., swim cap), and reinforcers (e.g., beads, tablet with music)	Wrist cuffs (both hands; baseline sessions only), blocking pads
	Chair Context	Padded session room with client on bench and one staff on either side of him. Later moved to client sitting in inflatable furniture with two staff on either side of him	Laptop computer, eye gaze device, bench toothbrush, inflatable furniture, toothpaste, competing items (e.g., swim cap), handwarmer reinforcers (e.g., beads, tablet with music)	Blocking pads
	Transition Context	Various clinical spaces	Laptop computer, competing item, handwarmer, cane	Blocking pads

## Data Availability

All accessible data are contained in the manuscript.

## Notes

1 This condition is typically referred to as “no interaction” but given that blocking was included in our analysis, we described this as “minimal interaction”

2 Gerard could not independently remove the cuffs, so the 0.2% decrement in engagement reflects a lapse in the precision of data collection and not lack of engagement.

## Abbreviations

The following abbreviations are used in this manuscript:

A-CSA	Augmented Competing Stimulus Assessment
ASRA	Alternative Self-Restraint Assessment
CSA	Competing Stimulus Assessment
CVI	Cortical visual impairment
DRA	Differential reinforcement of alternative behavior
FA	Functional analysis
FR1	Fixed ratio 1
FCR	Functional communication response
RPM	Responses per minute
SIB	Self-injurious behavior
VI2	Variable interval 2
VR5	Variable ratio 5
